# Understanding structural and molecular properties of complexes of nucleobases and Au13 golden nanocluster by DFT calculations and DFT-MD simulation

**DOI:** 10.1038/s41598-020-80161-z

**Published:** 2021-01-11

**Authors:** Ghazaleh Hashemkhani Shahnazari, Masoud Darvish Ganji

**Affiliations:** 1 Department of Organic Chemistry, Faculty of Pharmaceutical Chemistry, Tehran Medical Sciences, Islamic Azad University, Tehran, Iran; 2 Department of Chemistry, Qaemshahr Branch, Islamic Azad University, Qaemshahr, Iran

**Keywords:** Computational chemistry, Density functional theory, Molecular dynamics, DNA and RNA

## Abstract

The characterization of the complexes of biomolecules and nanostructures is highly interesting and benefits the rational development and design of nano-materials and nano-devices in nano-biotechnology. In this work, we have used dispersion corrected density functional theory (DFT**-**D) as well as DFT based molecular dynamics simulations to provide an atomistic understanding of interaction properties of DNA nucleobases and Au13 nanocluster. Various active sites of interacting molecules considering their relative orientation and distance are explored. Our goal is to stimulate the binding characteristics between two entities and evaluate this through the interaction energy, the charge transfer, the electronic structure, and the specific role of the molecular properties of the nucleobase–Au13 system. The primary outcomes of this comprehensive research illuminated that nucleic bases have potent affinity for binding to the Au cluster being chemisorption type and following the trend: Adenine > Cytosine > Guanine > Thymine. The AIM analysis indicated that the binding nature of the interacting species was predominantly partial covalent and high polar. We discuss the bearing of our findings in view of gene-nanocarrier, biosensing applications as well as nanodevices for sequencing of DNA.

## Introduction

Over recent decades, the emerging science of theranostic nanomedicine has advanced therapeutic paradigm significantly. Nanomedicine can provide an early and highly accurate diagnosis of clinical symptoms and effective treatments without side issues^1–3^. Multifunctional nanostructured materials play a pivoting role in the development of this science in various aspects such as initial diagnosis, gene, and drug delivery, imaging agent, tissue engineering, and monitoring of therapeutic responses with a notable potential for the treatment of a wide spectrum of diseases in almost all branches of medicine include immunology, oncology, ophthalmology, pulmonary, cardiology, endocrinology, dentistry, and orthopedic^4–7^. Different nanostructures have presented magnificent promise for target-specific delivery of genes and drugs in the body which offer unique strategies in different diseases prevention, diagnosis, and therapy. Nanoparticles are mainly composed of organic nanoparticles such as dendrimers, liposomes, polymeric micelles, and carbon nanostructures, and also inorganic nanoparticles namely elemental metals, metal oxide, etc.^8–10^. Among various inorganic nanoparticles, gold nanomaterials such as gold nanoparticle (AuNPs) and gold nanoclusters (AuNCs) have been found to be highly efficient for biomedical applications include immunoassays and genomic; photothermolysis and detection of cancer tumorigenic cells; biological sensing; monitoring of cells and optical bioimaging; photodynamic therapy of pathogenic microorganisms; wound healing; and target-specific delivery of drugs, DNA, peptides, and genes. Gold nanoclusters (AuNCs) as essential multifunctional inorganic nanoparticles have represented remarkable physicochemical properties for delivery applications^11–16^. AuNCs are versatile quantum-sized gold nanoparticles that are composed of very few numbers of atoms (less than 250 atoms per nanocluster). The core diameter of AuCNs ranges from the subnanometer to 2 nm and this nanocluster exhibits tunable ultrasmall size structure, high biocompatibility, inertness, and distinguished chemical stability. AuNCs have an ultrahigh surface area to volume ratio and surface that can be steadily modified with various ligands comprising of functional groups that illustrate high affinity for gold surfaces such as amines, phosphines, and thiols. Utilizing the functional groups to be anchored to the ligands, subordinate segments e.g. antibodies, proteins, oligonucleotides, etc., can create a broad range of functionalities in living cells^17–22^. The development of such gold nanoconjugate has rendered many possible strategies for different challenges including detection techniques, bioelectronics, precise organization of nanoparticles into dimer and trimers with variable shapes and sizes onto DNA templates, engineered assembly, and crystallization of particles. AuNCs are noteworthy structures for nucleic acid delivery. Nanoplatforms of Au**-**DNA/RNA conjugate systems have been studied in many researches as high potent agents for the delivery of a wide range of biospecies^23–27^. Alexander et al. investigated in AuNC-DNA nanoconjugate system for anticancer drug delivery^28^. In this respect, doxorubicin (DOX) was loaded to a nanoconjugate system containing DNA-capped AuNCs for task-specific DOX intercalation which inhibits topoisomerase II and prevent cancer cell growth. Drug binding was affirmed via monitoring the maximum of AuNC plasmon resonance, DNA melting temperature, and enhancement in hydrodynamic radius as a function of [DOX]/[DNA] ratio. Moreover, the final capacity of drug release to the target DNA was studied thoroughly. Yan et al. developed a novel nanoplatform of 3D superstructure based on the origami folding and growth of DNA on AuNPs for molecular transporting and unloading in cellular delivery applications and imaging^29^. They took the advantages of the rigidity of AuNPs and the flexibility of DNA nanostructures simultaneously. They realized that this 3D superstructure has a high capacity for molecule-loading. Vankayala et al. developed a novel strategy of nucleus-targeting using multifunctional AuCNs**-**based theranostic nanoplatform which is capable for performing gene delivery, fluorescence imaging, and photodynamic therapy for the devastation of cancer cells simultaneously^30^. Ju et al. reported the self-assembly of AuCNs with SpCas9 protein as a useful nanoconjugate system for high efficient delivery of SpCas9 to the cell nucleus at the physiological circumstance^31^. They realized that this nanoconjugate system is highly dependent on pH and has more stability at higher pH but can be disassembled at negligible pH. The process of assembly-disassembly simplifies the delivery of SpCas9 in to cell and consequently to the cell nucleus. Kim et al. surveyed about gene delivery application of nanoconjugate system of single stranded DNA and AuNPs for oligo antisense DNAs target-specific to any desired gene without any pernicious effect on ordinary cell physiology^32^. The deduced that this nanoconjugate system can be simply used for the delivery of various biospecies such as ribozyme, siRNA, DNAzyme, nucleotide acid, etc. According to the literature, numerous investigations have been earmarked to the delivery applications of nanoconjugate system of Au-based nanostructured materials and DNA^33–36^. The interaction between Adenine base and Au13 cluster was studied by using density functional theory (DFT) method^37^ and the results demonstrated the strong bonding of host–guest complex. Dabhi et al.^38^ investigated the interaction between nucleobases and boron nitride nanoribbons (BNNRs) by dispersion corrected DFT calculations. The estimation of adsorption energies showed that zigzag and armchair BNNRs, respectively, follow the following orders of adsorption pattern with the nucleobases: G > C > A > T > U and G > T > A > U > C. They also found that the origin of the binding was the π–π stacking.

Despite surveys that have been carried out on the interaction between DNA and its bases and small gold clusters including three to four bonded gold atoms, there is still lacking of a principled study on interaction of nanocage form of gold nanoclusters. It was concluded from literature that gold nanoparticles or nanorods^39^ can bind with DNA base molecules and other biological molecules^40^ and form aggregates inside the cells. So knowing how the binding of gold nanoclusters with DNA bases molecules and the changes in chemical and physical properties at ambient conditions would be necessary. This work represents valuable information about the nature of interactions and the effect of the environmental condition (solvent) on the interactions for their application in molecular electronics, biotechnology, and nanotechnology.

Herein, we have investigated comprehensively the interaction of nucleic acid bases namely adenine (A), cytosine (C), guanine (G), and thymine (T) with the Au13 nanocluster by using DFT calculation. Nucleobases are nitrogenous biological compounds existed in fundamental structure of nucleotides in DNA and RNA and play an increasingly essential role in different biological fields particularly in therapeutic applications^41,42^. To this purpose, adsorption properties, binding affinities, and electronic properties of nucleobases functionalized Au13 were studied using dispersion corrected DFT calculation as a modern computational approach for molecular systems. The main aim of this project was dedicated to identify an appropriate biospecies deliverer on the basis of AuNCs. In this study, the molecular structures corresponding to their elemental length and bonds were modeled and then, the density functional theory (DFT) calculations as well as DFT-based molecular dynamics (MD) simulations were carried out to calculate the different interaction parameters related to the nanoconjugate systems containing nucleobases and AuNCs. Our initial outcomes revealed that there is a potent affinity of bases for binding to the Au cluster regarding the following order A > C > G > T. The calculated interaction energies indicated that interaction nature was typical for the chemisorption. Moreover, the AIM analysis divulged that the binding nature of the interactions is highly polar and partially covalent. Both utilized methods, namely, DFT-MD simulation at ambient circumstances and DFT-D3 exhibited that nucleobases tend to form a stable complex with Au13. It should be noted that in the previous work^37^, only the interaction of Adenine base with Au13 cluster has been investigated with the different computational approach. Furthermore, the selected molecular systems were in the gaseous phase while in the present study we have considered the explicit water molecules surrounding the whole host–guest complexes. In addition, the state-of-the-art DFT-MD simulation employed in this paper can mimic the ambient conditions that differs significantly with the previous researches carried out up to now. Elaborated information about computational methodology of this project and the discussions on achieved results are presented in the next sections.

## Computational details

All calculations were carried out on the basis of DFT method to study the interaction between the considered nucleobases including adenine, cytosine, guanine, thymine, and uracil and Au13 golden cluster. DFT is the main computational technique, which extensively utilized in this research. Quantum-based designs were conducted using ORCA (3.0.3)^43^ and all electrons DFT calculations were investigated in accordance with the revised Perdew–Burke–Ernzerhof (revPBE) functional and generalized gradient approximation (GGA)^44–46^. To achieve this objective, the geometry of designed structures was optimized and energy calculations were respectively carried out for all atoms utilizing triple**-**ζ plus polarization basis set (def2**-**TZVP) in accordance with the method suggested by Ahlrichs et al.^47–49^. For the transition metals atoms (Au), we used the [SD (60, MDF)] scheme to describe the effective core potential (ECP)^50,51^. Accordingly, the normal optimization convergence criteria have been satisfied using ORCA and this process was considered to thoroughly optimize all structures for SCF iterations set to VeryTightSCF to diminish the contaminating of noise in the gradient calculations. In order to examine the long-range dispersion interactions, Grimme’s atom pair-wise dispersion corrections, 3rd version, with Becke–Johnson damping, called D3**-**BJ, were combinationally employed^52–54^. The conductor-like screening models (COSMO)^55^ scheme was utilized to describe implicitly the electrostatic interaction of molecules with solvent (solvent effects).

The influence of the imperfect nature of the basis set in non-covalently bonded systems was reduced whereas the basis set superposition error (BSSE) is decreased utilizing counterpoise correction^56^. On the other hand, the covalently bonded systems were investigated when the optimized structures were subjected to single point energy calculations on the basis of def2-TZVP basis set until the energies were approached to the basis set limit. Energies of adsorption were calculated by the following equation:

1 $$E_{{{\text{ads}}}} = E_{{{\text{Au13}}}} -_{{{\text{NB}}}} - \, \left( {{\text{E}}_{{{\text{Au13}}}} + {\text{ E}}_{{{\text{NB}}}} } \right) \, - \sigma_{{{\text{BSSE}}}} ,$$

where *E*_Au13_**-**_AA_, *E*_Au13_, and *E*_NB_ are respectively related to the total energies of the complex, golden cluster Au13, and nucleobases.

The quantum theory of atoms in molecules (QTAIM) method, well-known as AIM theory, was employed to study and determine the nature of the interaction of complexes. The theoretical foundation of AIM theory has been explained in related literatures^57–59^. To achieve this objective, the ORCA code was employed to acquire the wave-function utilized in the bonding analysis applying the DFT method. The topological analyses and the evaluation of local characteristics properties were conducted utilizing the Multiwfn program package^60,61^.

A bonding critical point (BCP) in accordance with the AIM theory locates between two atoms containing the considered bond. Hence, the electron density (ρ(r)) and the sign of its Laplacian can be determined whereas the considered bond is covalent or ionic electrostatic one as an agreement based on the sign of $\nabla^{2} p(r)$. In other words, the negative amount of $\nabla^{2} p(r)$ ($\nabla^{2} p(r)$ < 0) is considered when the covalent bond is present and the $\nabla^{2} p(r)$ > 0 is contrastingly considered if a closed shell (electrostatic) interaction exists. By considering the shared interactions, the electron density can be distributed through the distance linking the relating nuclei whereas the accumulation of charges can appear for the closed-shell interactions at the terminal nuclei. Accordingly, the BCP in the intermediate situation of the bond should be calculated based on the depletion of electron density.

The total energy density ($H(r_{BCP} )$) to the Laplacian of the electron density in accordance with the Bader’s theory as following^58^:

2 $$1/4\nabla^{2} \rho (r_{BCP} ) = G(r_{BCP} ) + H(r_{BCP} ),$$

where G(r_BCP_) is related to the kinetic energy density, which constantly has a positive value.

The molecular properties including gap energy (*E*_*g*_), hardness (*η*), and softness (*s*) for the related species and their interactions were calculated utilizing the following equations.

3 $${\text{I }} = \, - {\text{ E}}_{{{\text{HOMO}}}} ,$$

4 $${\text{A }} = \, - {\text{ E}}_{{{\text{LUMO}}}} ,$$

5 $$\eta = { 1}/{2 }\left( {{\text{I}} - {\text{A}}} \right),$$

6 $$S = \, \left( {{1}/{2}} \right) \, \eta ,$$

7 $$E_{g} = {\text{ E}}_{{{\text{LUMO}}}} - {\text{ E}}_{{{\text{HOMO}}}} ,$$

where HOMO and LUMO are related to the highest occupied molecular orbital and the lowest unoccupied molecular orbital states of energy transitions.

We have also carried out DFT-MD simulation using the SIESTA (Spanish Initiative for Electronic Simulations with Thousands of Atoms) software^62^ that pays the Born–Oppenheimer dynamics approximation for molecular systems simulations. The norm-conserving Troullier–Martins pseudopotential^63^ was used to describe the electrons and ions interactions. The GGA-PBE is employed to describe the exchange–correlation potentials. We used the localized atomic orbitals for valence wave functions and double-ζ polarized (DZP) basis sets with a cutoff energy of 125 Ry for molecular systems under consideration. The Velocity Verlet^64^ algorithm was utilized for solving Newton’s equations of motion. We employed the NVT ensemble by using Nosé−Hoover^65^ thermostat to maintain the temperature at ambient condition (300 K). A *k*-points meshes of 1 × 1 × 1 for *Monkhorst–Pack*, i.e.; gamma points, was used for the simulation of molecular system under study. In order to describe the electrostatic interactions, the *Ewald sum scheme* was employed. The time step was set to 1.0 fs and the total simulation time steps was 8.5 ps.

## Results and discussion

The production and utilization of functional materials at the nanoscale have raised concerns of sciences and technologies. As a role of thumb, the interaction nature of interacting systems and their interface region give helpful data in the fabrication of appropriate nanomaterials. In order to gain a deep understanding of the interaction characteristics of DNAbases interacting with the Au13 cluster and the influence of solvent and environment conditions on the complexes under study, we have performed DFT-D3 calculations in the gas phase implicit solvent scheme and explicit solvent media and also DFT-MD simulations. Specifically, we will evaluate the interaction of Au13/DNA bases including water molecules at room temperature.

Firstly, interactions of the Au13 cluster with DNA bases are investigated with the framework of the DFT-D3/revPBE method by exploring the variety of interaction sites. Next, Au13-bases complex embedded in the explicit water molecules is evaluated with a similar computations approach. Since a truthful determination of the structural geometries of a nucleic base interacting with a gold cluster in solution media is a really difficult task due to the presence of competing entities finally the interaction between Au13 and nucleic base surrounded with water molecules is simulated by DFT-MD simulations at ambient conditions.

### Interaction between Au13 cluster and nucleic bases

**Figure 1:**
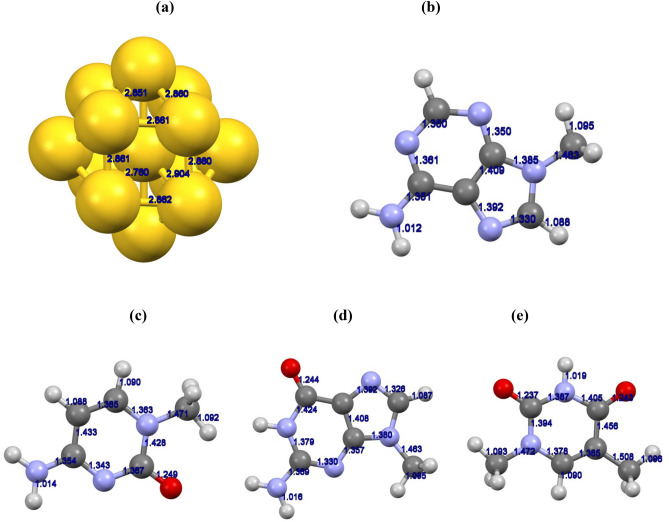
Optimized structures of (**a**) Au13 cluster, (**b**) adenine, (**c**) cytosine, (**d**) guanine, and (**e**) thymine bases with DFT**-**D3/TZVP.

In in this section, we will present the main outcomes of our performed investigation as well as relevant analyses by exploring the magnitude and type of the bond formation. The optimized geometry structures of the Au13 cluster and also nucleic bases (adenine, cytosine, guanine, and thymine) are represented in Fig. 1. The optimized structures utilized in the optimization of DNA**-**Bases/Au13 complexes, were obtained by approaching the nucleic bases toward the active sites of the golden cluster through their potential active sites. It should be noted that each of the nucleic bases was located on various sites of the Au13 surface in various relative orientations. For instance the considered active sites of interacting entities, for the adenine molecule and the Au13 cluster, are represented in Fig. 1. For each configuration, the structures of both the Au13 cluster and the nucleic base were allowed to be fully optimized. Then, the interaction energy (*E*_int_) of each complex was calculated with the DFT**-**D3/TZVP model of theory by the related relation (Eq. 1) considering the BSSE corrections. The estimated interaction energies of energetically favorable configurations (the configuration with highest interaction energies, the most negative values, between selected orientations) for Au13/nucleic bases are listed in Table 1.

**Table 1:** The calculated interaction energies in gas and solvent media and solvation energies as well as charge transfer by Mulliken and Hirshfeld methods for nucleobases interacting with Au13 cluster.

Complex	*E*_int_ (eV)	*E*_int_ (eV)	*E*_solv_ (eV)	Q_T_ (*e*)	Q_T_ (*e*)
	Aqueous	Gas		Mulliken	Hirschfeld
Adenine/Au13	− 1.97	− 1.79	− 0.796	0.465	0.503
Cytosine/Au13	− 1.86	− 1.55	− 0.937	0.389	0.439
Guanine/Au13	− 1.69	− 1.50	− 0.411	0.313	0.371
Thymine/Au13	− 1.36	− 1.33	− 0.610	0.263	0.135

**Figure 2:**
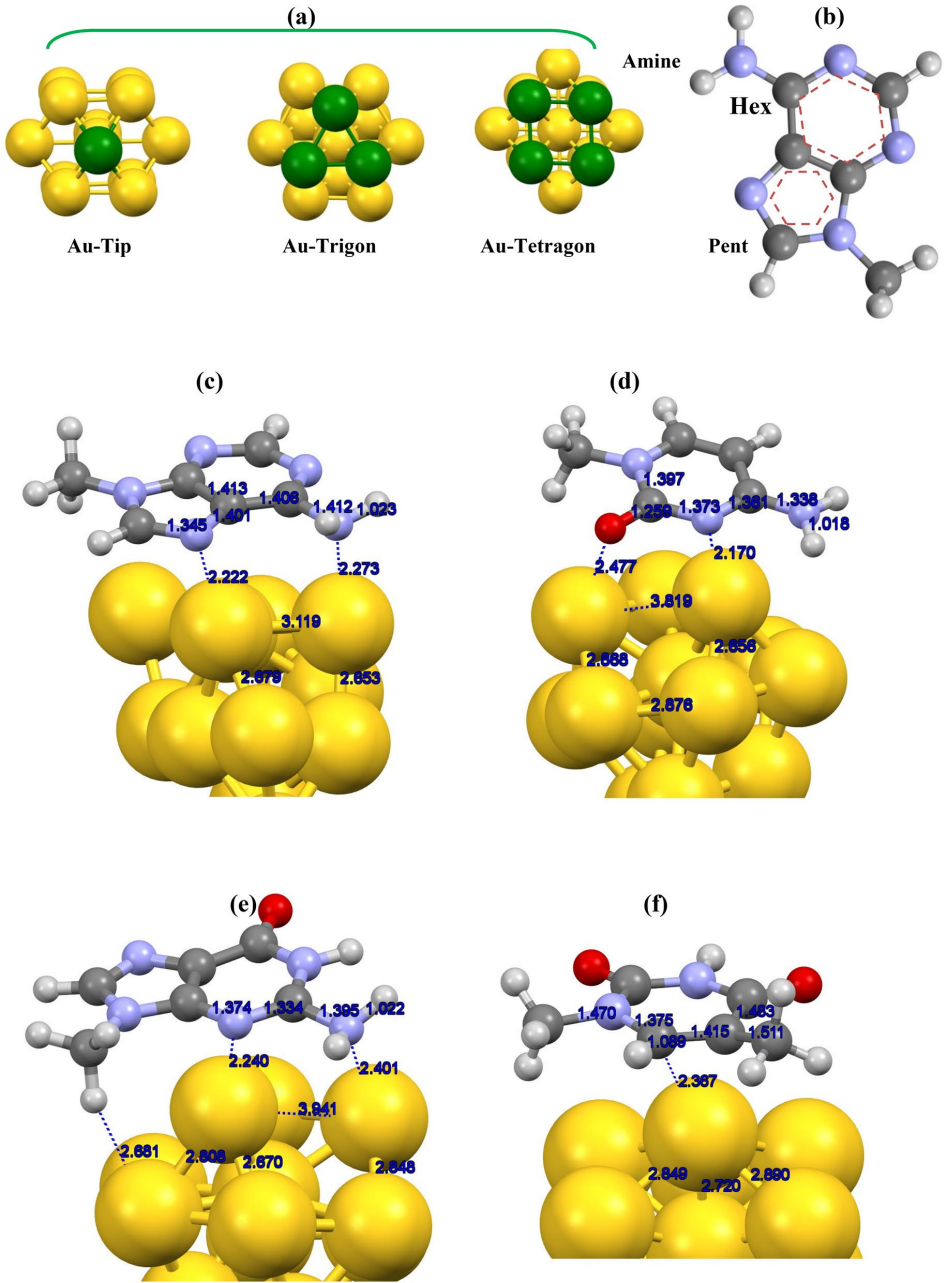
Representation of potential active sites for (**a**) Au13 cluster and (**b**) adenine. Optimized structures of (**c**) adenine/Au13, (**d**) cytosine/Au13, (**e**) guanine/Au13, and (**f**) thymine/Au13 complexes with DFT**-**D3/TZVP and implicit solvent media.

According to achieved outcomes, in all studied cases, a strong interaction happened between Au13 and nucleic bases with *E*_int_ of around − 30 to − 40 kcal/mol. These values agree with measurements of experiments for similar complexes (adenine/Au cluster)^66–69^. The magnitude of the interaction energies is relatively in the range of chemical interactions^70–74^. These strong interactions are explicable from considered structural geometries where the nucleic base's orientation is such that the polar groups (oxygen/nitrogen atom) are nearest to the gold atom of Au13 surface (see Fig. 2). In a dissimilar case, the interaction of thymine was found to be through the carbon atoms of the ring. Adenine with − 1.97 eV, has the most negative *E*_int_ and thus the highest binding affinity to the Au13 cluster whilst thymine with − 1.36 eV demonstrates the lowest affinity for the golden cluster. Moreover, cytosine and guanine with − 1.86 and − 1.69 eV are next in line after adenine. The strength of *E*_int_ varies for the four bases and follows the following trend:

$${\text{Adenine }} > {\text{ Cytosine }} > {\text{ Guanine }} > {\text{ Thymine}}{.}$$

Figure 2 represents the optimized structures along with equilibrium distances for the highest interaction configurations of the nucleic base/Au13 complexes. The bonding distance of nitrogen atoms of the adenine from the gold atoms of Au13 skeleton is estimated to be 2.27 Å (amine group)/2.22 (pentagon ring) Å, whereas for the guanine, the corresponding calculated distances are about 2.40 Å (amine group)/2.24 (hexagon ring) Å. The obtained bond lengths are close to previous theoretical values for similar systems^39,69^. The estimated average bonding lengths values between Au and N atoms with B3LYP/6-31G (d) [LANL2DZ] model of theory in adenine + (Au)n = 3, 6, 9, 12] complexes were varied about 2.2–2.6 Å^67^ that consistent to our findings with revPBE-D3/TZVP for Au13/adenine complex.

**Figure 3:**
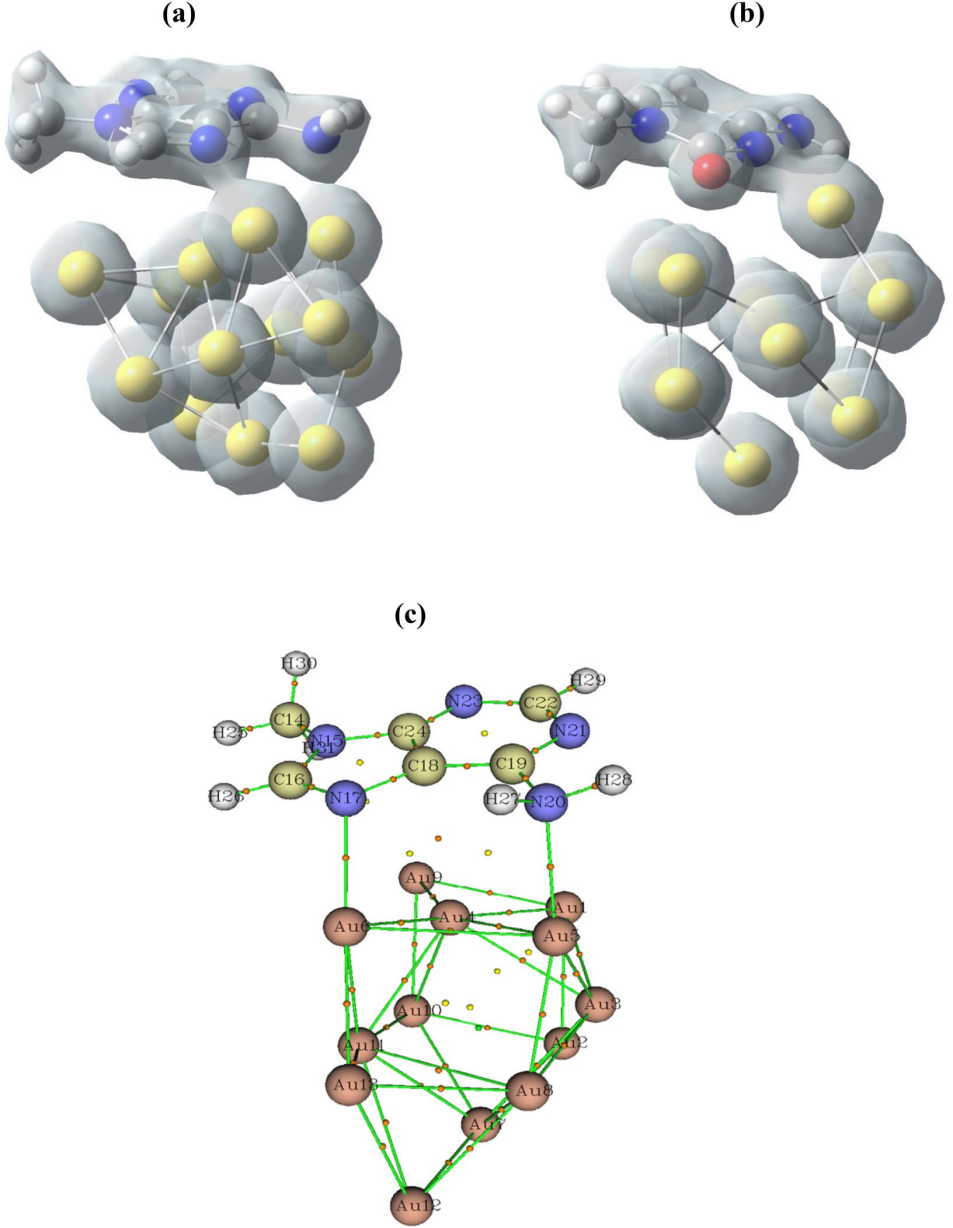
Calculated total electronics charge for (**a**) adenine/Au13 and (**b**) cytosine/Au13 systems with DFT**-**D3/TZVP (iso-value was set to 0.07). (**c**) Calculated bonding critical points (BCPs) for adenine/Au13 system with DFT‒D3/TZVP.

To explore the nature of the interaction and also the electronic properties of interacting systems, the charge density isosurface plots which demonstrate redistribution of electronic charges upon the interaction of nucleobases were calculated. The obtained isosurface plots for the adenine/Au13 and cytosine/Au13 complexes as the most stable complexes are depicted in Fig. 3a,b, respectively. Taking the favorable configurations of considered complexes, it can be found from the plots that slight redistribution of charges has occurred after the binding process. Furthermore, different accommodation pattern of electronic charges around the Au‒N and Au‒O bonds in two selected complexes was observed which indicates different bonding strength and also the electrons transfer phenomenon upon the adsorption. Particularly, changes introduced in the hybridization of involved bonds during the adsorption process, e.g.; the N atom of pentagon ring that re-hybridized from *sp*^2^ to *sp*^3^, facilitates the charge transfer between interacting molecules and accordingly, the golden nanocage was polarized, as depicted in the figure. Therefore, this polarization causes strong electrostatic attractions accompanied by the formation of covalent bonds between the nucleobases and the golden cluster. Mainly govern the overall interactions in this system. This finding can be approved by the charge analysis results as listed in Table 1 which point out the charge of about 0.5 *e* were transferred from the adenine/cytosine to the Au13 moiety. Furthermore, the charges of about 0.439, 0.371, and 0.135 e were transferred from the Au13 cluster to the cytosine, guanine. Meanwhile, the Hirshfeld analysis indicates that partial charges of the N atoms of the adenine which involved in the interaction with Au13 cluster, changed from − 0.621 (N**-**ring) and − 0.946 (N**-**amine) to − 0.166 and − 0.085, respectively.

The calculated energy gap which defines the difference between the HOMO and the LUMO states, as presented in Table 2 showed that the pristine Au13 cluster exhibits semi**-**conducting behavior with 1.434 eV gap energy and its semi-conducting property was maintained after the nucleobases adsorption. Strictly speaking, the obtained values of the HOMO–LUMO gap for the Au13 cluster remains almost unchanged upon the adsorption of nucleobases and reveals that the strong interactions of the host–guest complexes have a minor effect on the electronic behavior of the considered adsorbent.

**Table 2:** Molecular properties of nucleobases and nucleobases**/**Au13 complexes.

Molecule	Molecular properties						
	E_HOMO_	E_LUMO_	Hardness (η)	Softness (S)	Dipole momentum (μ)	Electron affinity (ω)	Gap energy (E_g_)
Au13	− 5.129	− 3.694	0.717	0.697	− 4.411	13.569	1.434
Adenine	− 6.166	− 0.921	2.622	0.191	− 3.543	2.394	5.245
Aden/Au13	− 4.652	− 3.093	0.779	0.641	− 3.872	9.617	1.560
Cytosine	− 6.450	− 1.129	2.660	0.188	− 3.790	2.699	5.321
Cytos/Au13	− 4.689	− 3.307	0.686	0.343	− 3.998	11.645	1.373
Guanine	− 5.949	− 0.697	2.626	0.194	− 3.323	2.102	5.253
Guan/Au13	− 4.685	− 3.320	0.682	0.733	− 4.003	11.739	1.365
Thymine	− 6.470	− 1.384	2.543	0.197	− 3.927	3.033	5.086
Thym/Au13	− 4.900	− 3.575	0.666	0.755	− 4.238	13.479	1.324

Moreover, the molecular properties of nucleobases interacting with the Au13 cluster were examined and the obtained values are tabulated in Table 2. The estimated hardness of the nucleobases in accompany with their gap energies were decreased while the softness and also their reactivity contrarily showed increasing values after the adsorption. According to achieved data, the greatest softness and hardness belong to thymine/Au13 and adenine/Au13, respectively. whilst the least softness and hardness belong to cytosine/Au13 and thymine/Au13, respectively.

Indeed, the enhancement of reactivity of the host–guest complex gives rise to the efficient loading performance of designed nanomaterial. Hence, the Au13 cluster can be chosen as a suitable nanomaterial for drug and gene delivery applications. Moreover, the performed studies improved our knowledge against the formation of probable complexes between biomolecules and nanostructures. This fact can be further studied in the fields of drug and gene delivery and relevant subjects based on nanomaterials.

In addition, the stability of the host–guest systems was evaluated by estimation of the solvation energy, *E*_solv_, of complexes under study. To this aim, full structural optimization with the same computational procedures was carried out for the energetically most stable configurations in the gas phase and then the solvation energies were determined. The following equation was utilized for the *E*_solv_ calculation:

8 $$E_{{{\text{solv}}}} = E_{{{\text{solvent}}}} {-}E_{{{\text{gas}}}} ,$$

where the pointed out terms indicate the solvation energy of the host–guest system, the total energy in the aqueous media, and the total energy in the gas phase, respectively. The calculated solvation energies are listed in Table 1. According to the achieved data, the complex of cytosine/Au13 possessed the greatest solvation energy of about − 0.937 eV compared to other created complexes, and guanine/Au13 possessed the least solvation energy of about − 0.411 eV. Further, the complexes of adenine/Au13 and thymine/Au13 with solvation energies of about − 0.796 and − 0.610 eV were next in line after cytosine/Au13. It can be found from the obtained *E*_solv_ with revPBE-D3/TZVP that, for all the complexes, the adsorbed nucleobase on the Au13 nanocage with negative values improved the solubility of the complexes within the solvent environment. Meanwhile, among the determined solvation energies for nucleobases, the cytosine demonstrated a larger value rather the other bases which indicate that this nucleobase possesses more affinity to be functionalized by the golden cluster in the aqueous solution.

### Interaction nature of Au13/Bases complexes

In this section, we present further explanations of regard to the nature of the bonding of the host–guest systems by using the AIMtheory analysis. To this aim, the bond critical points (BCPs) for optimized geometries of the host–guest system (adenine/Au13 complex) were calculated and listed in Table 3. Also, the BCPs parameters are demonstrated by the orange color in Fig. 3c. The calculated Laplacian, ∇^2^ρ, for the Au…N‒ring, and Au…N‒amine in the interaction regions were determined to be positive values of 0.256 and 0.229, respectively, which displays a rather strong depletion of charges at the considered critical point. On the other hand, the obtained negative values of the energy densities, *H*(r), for both selected bonds indicating the presence of an attractive interaction between involved atoms (Au…N). It should be noted that the negative values of *H*(*r*) and positive values of ∇^2^*ρ* demonstrates an intermediate state in bond formation in which a highly polar attraction accompanied by a partially covalent bond were occurred in the adsorption. Furthermore, the G(r)/V(r) parameters that give further insight into the bonding nature were estimated between 0.5 and 1 which indicates a polar covalent bond between interacting molecules. It was found that the G(r)/V(r) ratio for Au…N‒ring was larger than that the Au…N‒amine which indicates that there is stronger interaction in the former bond. To verify the obtained results for the interacting molecules, the BCPs parameters for the C−N and Au−Au bond in adenine and gold cluster were also calculated. We found negative values of ∇^2^ρ and H(*r*) for C−N bond that indicate the formation of a covalent bond between C and N atoms which agrees well with experiment and other theoretical reports for similar systems^75^. However, the estimated BCPs values for the Au−Au bond in Au13 cluster show positive value for ∇^2^ρ and negative value for H(*r*) that demonstrates a polar bond with partially covalent nature for adjacent Au atoms in the nanocage.

**Table 3:** Calculated BCPs parameters with DFT‒D3**/**TZVP for adenine/Au13 complex.

	BCP1 (Au6…N17)	BCP2 (Au5…N20)	BCP (Au8…Au12)	BCP (C16…N17)
$\uprho$(r)	0.827	0.755	0.532	0.344
${\nabla }^{2}\uprho (\mathrm{r})$	0.256	0.229	0.122	− 0.109
$H$(r)	− 0.182	− 0.154	− 0.968	− 0.492
G(r)	0.822	0.727	0.403	0.219

In a novel experiment, Lindsay et al.^64^ have explored the adsorption of the DNA bases on the Au (111) surface through the atomic force microscopy (AFM) and cyclic voltammetry (CV). They reported that the adenine, guanine and cytosine were adsorbed on the gold surface but thymine did not adsorb on the surface. The reported bond lengths values between the gold atoms are in good agreement with the earlier study^64^. Princia Salvatore et al.^74^ have studied the adsorption of the nucleobases (A, C, G and T) on the Au (110)-electrode surface using electrochemically controlled scanning tunneling microscopy (ECSTM) and CV as well as DFT calculations. They observed that the adsorption of all the DNA bases are on the gold surface were typical for the chemisorption. Furthermore, DFT calculations confirmed that adsorption was accompanied by partial transfer of charges from the bases to the gold surface resembling coordination chemical bonding. In order to examine the possibility of formation of complexes between bases and Au13 cluster studied in the present work, it is suggested to employ the convenient techniques such as ECSTM, AFM and CV.

### Effect of explicit solvent on the interaction properties

**Figure 4:**
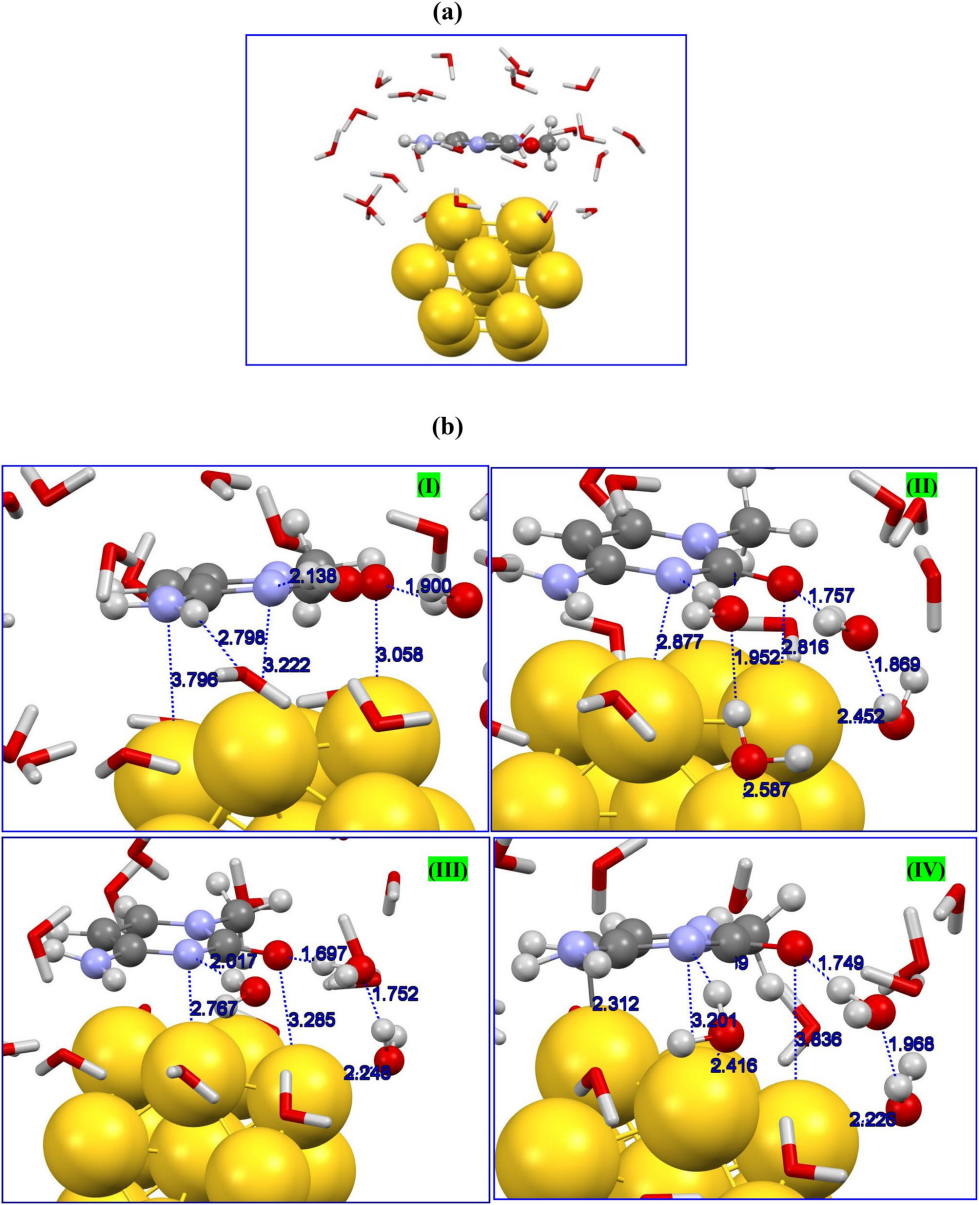
Schematic representation of (**a**) initial configuration and (**b**) snapshots of configurations during the optimization procedure for cytosine**-**Au13/25H_2_O system with DFT‒D3/TZVP.

In order to closely examine the possibility of formation of a complex between considered golden nanocluster and nucleobases and its application in bio-related fields we now simulate the host–guest system in media that almost mimics an environmental condition. It is necessary to reflect the role of solvents on the nature of the nature of interacting entities especially bio-molecules. Although the interactions of biological systems such as base pairs or folding of proteins can be studied sufficiently widespread in the gaseous phase, nevertheless, these activities do not oblige for the interactions of biomolecule interfaces as well as interactions with inorganic surfaces. This can be attributed to this fact that the above mentioned interactions are strictly environment-dependent. Accordingly, it is important to consider the real medium for bio-molecules under study to find truthful results and the solvent effects cannot be flouted. In this regard and for the sake of cost-effective purpose, we investigate the interaction behavior of cytosine-Au13 cluster in a media involving 25 water molecules surrounding the whole complex as shown in Fig. 4a. Full structural optimization with revPBE-D3/TZVP(-f) theory model was performed for the cytosine-Au13/25H_2_O system. Our DFT-D3 calculations showed that in the early optimization steps the cytosine move towards the golden nanocage and placed about 2.816/2.877 Å far from the Au13 surface though its O/N atoms. However, after movement of optimization steps we found that both O and N atoms was pushed back by existed water molecules attached to the nanocage skeleton through strong hydrogen bond formed between O and N atoms of cysteine and H atoms of water molecules. Figure 4b depicts the schematic of geometry structures of complex under study. Finally, in contrast to the optimized structure with implicit solvent (COSMO model), cytosine prefers to bound to the nanocage through its C atom of ring. The notable change in adsorption scheme clearly arises out of the presence of water molecules that prevent the binding of cytosine to the Au13 surface due to the hydrogen bonding forces. This is surprising that hydrogen bonds dominates the chemisorption of nucleobases on the metallic surface which has not been discussed so far neither through the experiments nor by theoretical studies. Indeed, in existent aqueous media, the water molecules will progressively approach and surround the polar groups of these bio-molecules up to the direct contact between the host and guest moieties was decreased. It should be borne in mind that more rigorous studies are mandatory to be carried out by using molecular dynamics (MD) simulations at finite temperature to accurately explore the global minimum energy surface as well as interpret the phenomena behind these host–guest systems.

### DFT-MD simulation of cytosine-Au13/H_2_O system

**Figure 5:**
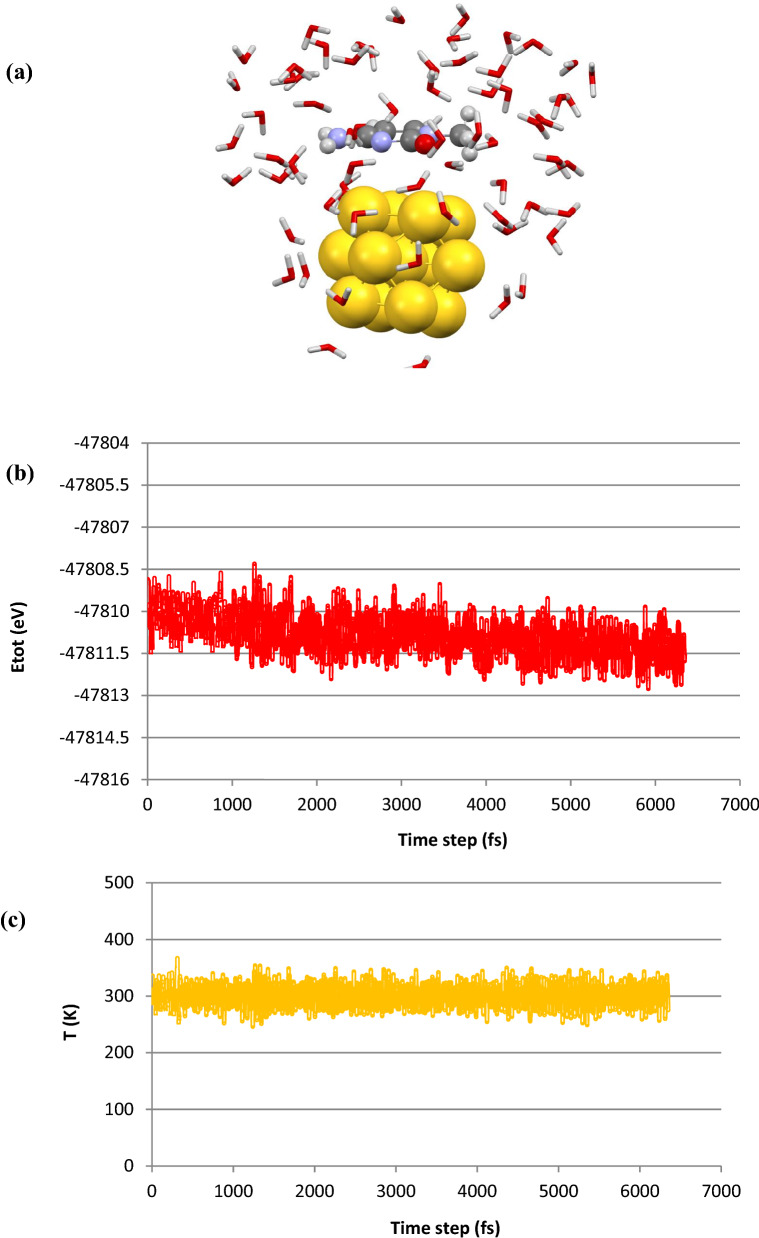

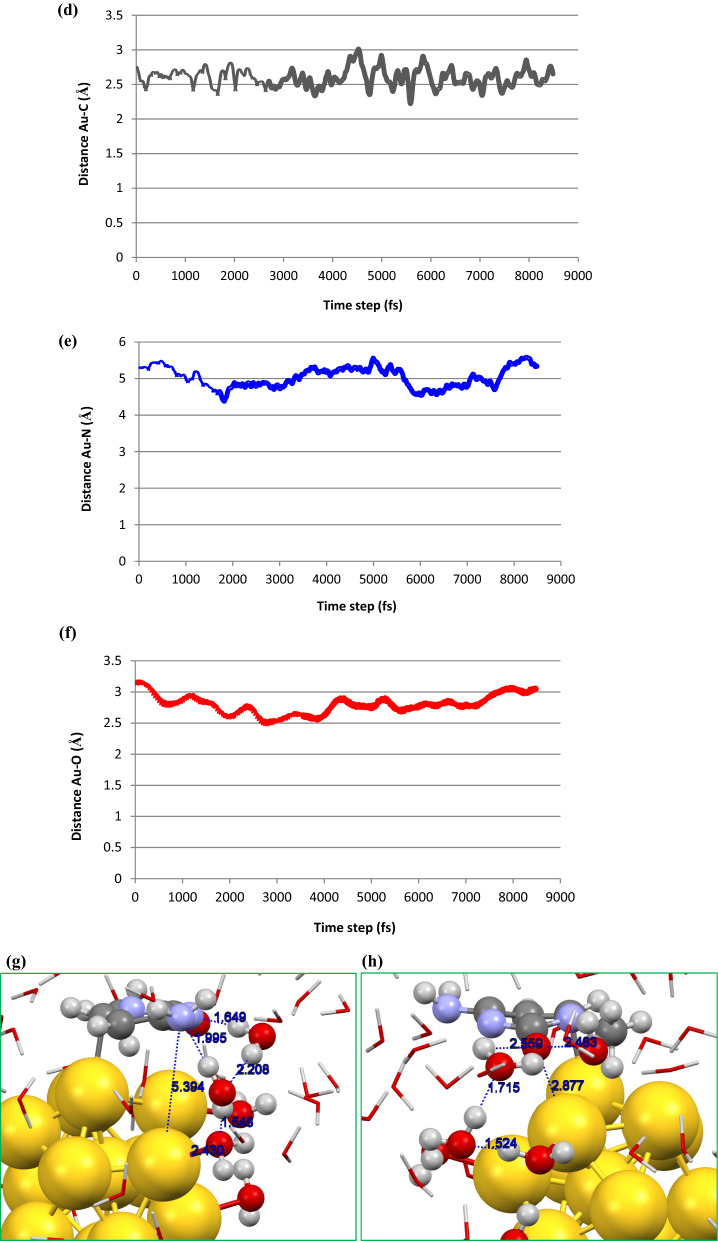
Representation of (**a**) initial model of cytosine/Au13/70H_2_O system considered for DFT**-**MD simulation. Calculated plots of (**b**) temperature and (**c**) total energy obtained during the DFT‒MD simulation procedure for cytosine-Au13/70H_2_O system. Snapshot of the molecular structure of cytosine-Au13/70H_2_O at 12 ps representing bond distances between water molecules and (**d**) NH_2_ and (**e**) O groups. Bonding distances between (**f**) C, (**g**) N, and (**h**) O atoms and nearest Au atom during the 8.5 ps of simulation times.

To give further insight into the stability of the host–guest complex in the presence of water molecules, the state**-**of**-**the**-**art DFT**-**MD simulation was performed at ambient conditions. By using DFT**-**MD simulation it is possible to carry out a realistic investigation on the interacting host–guest system considering bond forming/breaking of the complex at room temperature. For the moment, the transfer of charges between interacting moieties can be specifically analyzed. It should be noted that the MD simulation presents reasonably the global minimum criteria for complex systems. To perform a simulation procedure, we have considered a complex system consisting of a cytosine attached to the Au13 cluster surrounded by 70 water molecules, as represented in Fig. 5a. Then, we carried out DFT**-**MD simulation to evaluate the interaction nature as well as the stability of cytosine**-**Au13 cluster system in aqueous media. Our DFT**-**based MD simulation showed that the host–guest system reached to the thermodynamics equilibrium criteria as demonstrated by plotting the total energy of the whole system *vs.* the time steps (see Fig. 5b). The obtained plot by DFT**-**MD simulation at 300 K represents a sharp decrease of total energy at the starting simulation time steps while it reaches an average value of about − 47,811.5 eV at 4.5 fs. This finding demonstrates the stability of the molecular system after the pointed simulation time. Also, our simulation outcomes display the reasonable fluctuation of temperature around 300 K which indicates the equilibrium situation of the whole system during the simulation times (see Fig. 5c).

Our DFT**-**based MD simulation results showed that cytosine tends to attach to the gold cluster surface through its O and NH_2_ groups as well as C atom of the ring at the beginning of simulation times, about 2 ps for O and N atoms, as represented in Fig. 5d,e,f. Plots of bonding distances against simulation times showed the binding of the cytosine to gold cluster during the simulation procedure and a new bond forms between C atom of the ring and an Au atom (see Fig. 5d). However, one can find that NH_2_ and O groups gradually located far away from the cage surface during the simulation period, as can be seen from Fig. 5e,f. This can be attributed to the presence of water molecules that tend to be attached to the gold surface and drive back the N and O atoms of cytosine by strong hydrogen bonding forces and hence avoid the bond formation between O/N and Au atoms. The schematic representation of water molecules attached to/arranged around the gold cluster is given in Fig. 5g,h. It was found from these distances plots that cytosine wins the competition for binding to gold cluster rather than water molecules by its C atom of the aromatic ring and the formed host–guest complex remains stable thermodynamically at ambient conditions. Our DFT**-**MD simulation demonstrates that cytosine/Au13 cluster complex is energetically stable and hence Au13 cluster could be used as a suitable carrier/adsorbent for nucleic bases.

## Conclusion

In work, integration between base–Au13 cluster was investigated by dispersion corrected DFT based calculations within three strategies. First, we evaluated the interaction properties of host–guest molecules by DFT-D3/TZVP(**-**f) model of theory in implicit solvent media. It was found that the nucleic base’s affinity for binding to Au cluster follows the trend A > C > G > T. Base–Au13 interactions nature was studied by AIM analysis and showed that the interactions have a highly polar and partially covalent bonding nature. While the implicit solvent model showed the bonding formation between Au13 cluster and active sites of bases through N/O atom, the modeling of explicit water molecules revealed a different bonding pattern. Both DFT**-**MD simulation at ambient condition and DFT**-**D3/TZVP(**-**f) calculation indicated that cytosine prefers to bound to the cluster surface through its C atom of the aromatic ring. This is attributed to the strong hydrogen bonding between water molecules and the nucleic base. These simulation findings provide a thorough picture of base–Au cluster interactions and shed supplementary light on the mechanics of bio-functionalization of golden nanostructures.
